# Effects of Peripheral Visual Field Loss on Eye Movements During Visual Search

**DOI:** 10.3389/fpsyg.2012.00472

**Published:** 2012-11-05

**Authors:** Emily Wiecek, Louis R. Pasquale, Jozsef Fiser, Steven Dakin, Peter J. Bex

**Affiliations:** ^1^Massachusetts Eye and Ear InfirmaryBoston, MA, USA; ^2^Department of Ophthalmology, Harvard Medical SchoolBoston, MA, USA; ^3^Institute of Ophthalmology, University College LondonLondon, UK; ^4^Department of Medicine, Channing Division of Network Medicine, Harvard Medical SchoolBoston, MA, USA; ^5^Volen National Center for Complex Systems, Brandeis UniversityWaltham, MA, USA; ^6^NIHR Biomedical Research Centre for Ophthalmology, Moorfields Eye Hospital, NHS Foundation TrustLondon, UK

**Keywords:** eye movements, visual search, low-vision, glaucoma, peripheral vision, and natural scenes

## Abstract

Natural vision involves sequential eye movements that bring the fovea to locations selected by peripheral vision. How peripheral visual field loss (PVFL) affects this process is not well understood. We examine how the location and extent of PVFL affects eye movement behavior in a naturalistic visual search task. Ten patients with PVFL and 13 normally sighted subjects with full visual fields (FVF) completed 30 visual searches monocularly. Subjects located a 4° × 4° target, pseudo-randomly selected within a 26° × 11° natural image. Eye positions were recorded at 50 Hz. Search duration, fixation duration, saccade size, and number of saccades per trial were not significantly different between PVFL and FVF groups (*p* > 0.1). A χ^2^ test showed that the distributions of saccade directions for PVFL and FVL subjects were significantly different in 8 out of 10 cases (*p* < 0.01). Humphrey Visual Field pattern deviations for each subject were compared with the spatial distribution of eye movement directions. There were no significant correlations between saccade directional bias and visual field sensitivity across the 10 patients. Visual search performance was not significantly affected by PVFL. An analysis of eye movement directions revealed patients with PVFL show a biased directional distribution that was not directly related to the locus of vision loss, challenging feed-forward models of eye movement control. Consequently, many patients do not optimally compensate for visual field loss during visual search.

## Introduction

Peripheral vision provides critical wide field information about the environment (Millodot and Lamont, 1974; Thorpe et al., 2001; Velisavljevic and Elder, 2008; Larson and Loschky, 2009; Kwon and Legge, 2011), despite its low resolution (Westheimer, 1982). The coarse-scale encoding provided by peripheral preview confers functional benefits (e.g., faster reading speeds; Rayner et al., 2011) and serves to guide saccadic eye movements, bringing targets into the fovea for high-resolution inspection during periods of fixation. In a visual search task, such a process can be repeated until the desired target is located (for review, see Hayhoe and Ballard, 2005). Under some conditions, this searching oculomotor behavior may be driven by bottom-up “feature salience,” based on low-level sensory contrasts (Nothdurft, 1993; Wolfe, 1994; Itti and Koch, 2001; Mazer and Gallant, 2003) and information gain (Najemnik and Geisler, 2005). Additionally, top-down, task-demands may influence fixation behavior based on task-relevant information (Land et al., 1999; Foulsham and Underwood, 2007; Henderson et al., 2007; Einhäuser et al., 2008).

Glaucoma and disorders involving the formation of optic nerve drusen are prototypical diseases in which foveal acuity is preserved, but progressive optic neuropathy results in peripheral visual field loss (PVFL; Spalding, 2002). In the clinic, glaucoma patients frequently report difficulty with search-based tasks (Ramulu, 2009). Impaired performance on visual search tasks may be attributed to the lack of peripheral information available to patients with PVFL. Some investigators found PVFL leads to longer search times within natural images (Viswanathan et al., 1999; Green et al., 2002), as well as longer search times and higher error rates for displays composed of optotypes (Coeckelbergh et al., 2002). However, another group failed to replicate the findings of a difference with optotype displays (Smith et al., 2011), and Coeckelbergh et al. (2002) found no apparent accompanying change in oculomotor behavior, except for an increase in the number of saccades to previously inspected locations.

There is also controversy over the effect of PVFL on more naturalistic tasks. Crabb et al. (2010) asked glaucoma patients with PVFL to indicate hazardous events in a real-world movie used in a driving test. Patients made more saccades, fixations, and smooth pursuit eye movements per second than controls, and the mean fixation duration was shorter. In contrast, Luo et al. (2008) reported that PVFL patients and normal controls executed similar eye movements when performing visual search and real-world walking experiments, suggesting patients with PVFL did not adapt their oculomotor behavior to compensate for peripheral deficits.

The heterogeneity of pathological vision loss may contribute to the conflicting results reported in the literature. Consequently, a number of groups have simulated controlled visual field loss with gaze-contingent artificial scotomas (Crane and Kelly, 1983). Under these conditions, search times increase as the field of view contracts (Cornelissen et al., 2005) and when information is masked in the periphery but retained at fixated locations (Pomplun et al., 2001). These data are consistent with feed-forward models of eye movement behavior, in which potential targets for fixation are selected in the periphery. This organization implies that targets must fall closer to the fovea to be detected when the peripheral visual field is masked by an artificial scotoma (Najemnik and Geisler, 2005), with little influence from top-down knowledge of the layout of natural scenes.

The visual search stimuli used in many previous studies with real and simulated scotomas employed discrete targets on highly regular matrices. Such stimuli lack the rich array of features and structural variation normally present in natural scenes, and this redundancy in natural scenes generally serves to assist target detection in the periphery (Bruce and Tsotsos, 2009; Wolfe et al., 2011). Consequently, the oculomotor behavior supporting such search tasks may not be consistent with those obtained under natural viewing conditions. In the present study, we overcome these limitations by asking subjects to perform a visual search for a target embedded within a natural scene. Using this paradigm, we can analyze oculomotor behavior using a setting and task more akin to patients’ interactions with their everyday environment to better understand the interaction between central and peripheral vision in a visual search task.

We believe that such a naturalistic setting will emphasize the interplay between peripheral and foveal vision necessary to support search, and therefore hypothesized that PVFL patients’ absence of low resolution peripheral information will impair search performance under such conditions. We also hypothesized that if eye movements are guided by salient features of the image, then any undetected features (falling on the location of a visual field defect) will alter the predicted path of performance. Specifically there should be few eye movements into locations of PVFL. Alternatively, if search is guided by the demands of the task, knowledge of scene layout, and other top-down factors, then PVFL may not affect performance and oculomotor behavior will not differ significantly between patients and controls.

## Materials and Methods

### Observers

Participants were recruited from the Glaucoma Service at Massachusetts Eye and Ear Infirmary (MEEI) in Boston, MA, USA. Ten patients with PVFL and 13 reference subjects with full visual fields (FVF) were recruited. Participants included in the PVFL group were given the clinical diagnosis of glaucomatous optic neuropathy or optic nerve drusen with elevated intraocular pressure. Patients with optic nerve drusen were included because they develop optic-nerve-based PVFL similar to glaucoma (Spalding, 2002). All 10 observers had reproducible visual field loss consistent with nerve fiber layer dropout in at least one eye on reliable automated perimetric tests [24-2 Humphrey Visual Field tests using the Swedish Interactive Thresholding Algorithm (SITA)]. Tests were reliable if fixation loss was ≤33%, false positive rates were ≤20%, and false negative rates ≤20%. Participants included in the FVF group were glaucoma suspects with no visual field loss on at least two tests and did not have a history of IOP >21 mm Hg. All PVFL and FVF patients had acuity of 20/40 or better. Suspects were chosen as an ideal comparison group because they are under high surveillance for progression to manifest VF loss, and many other variables (e.g., IOP medication, age, familiarity with visual field testing) can be equated between the two groups. The mean age of the PVFL group and FVF group was 68.3 ± 13.6 and 64.8 ± 11.3 years, respectively (*p* = 0.5). Patient information is displayed in Table 1. This study was approved by the IRB committees of MEEI and Schepens Eye Research Institute and adhered to the tenets of the Declaration of Helsinki. All participants gave informed written consent before participating in the study.

**Table 1:** **Peripheral visual field loss patient information and demographics**.

Subject	Age	Gender	Diagnosis	Eye	Visual acuity	MD (db)	PSD (db)	*P* value (based on χ^2^ from FVF saccade direction distribution)	% Of positively correlated points	Standard deviations from FVF observer	Angle direction from FVF observer (°)
1	83	Female	POAG	L	20/30	−5.94	3.71	0.0001	68.63	1.46	68.15
2	82	Female	CACG	R	20/25	−6.24	5.01	0.0911*	69.23	1.21	86.88
3	82	Female	POAG	R	20/20	−4.42	2.77	<0.0001	74.51	2.25	83.38
4	58	Female	ON drusen with treated OHTN	L	20/20	−6.53	7.27	0.0001	33.33	1.04	157.37
5	55	Male	ON drusen with treated OHTN	R	20/20	−8.53	9.96	0.2308*	76.92	1.02	73.02
6	74	Female	Exfgl	L	20/40	−6.51	6.32	<0.0001	76.47	0.86	55.12
7	67	Male	POAG	L	20/20	−4.65	6.32	0.0001	58.82	0.95	57.55
8	79	Male	POAG	L	20/25	−13.3	4.57	0.0023	78.85	1.21	73.47
9	58	Male	Ang rec gl	L	20/25	−28.1	9.73	0.0006	80.39	6.49	79.35
10	45	Female	POAG	L	20/20	−9.03	13.43	0.0029	67.31	1.05	71.72

*Diagnoses: *ON drusen with treated OHTN*, optic nerve drusen with treated ocular hypertension; *Exfgl*, exfoliation glaucoma; *Ang rec gl*, angle recession glaucoma; *NTG*, normal tension glaucoma; *POAG*, primary open-angle glaucoma; *CACG*, chronic angle closure glaucoma. Best corrected visual acuity was not significantly impaired in any subject tested, but all patients had significant visual field defects. MD, mean deviation. PSD, pattern standard deviation. The χ^2^ significance indicates whether the distribution of eye movement directions was significantly different from full visual field (FVF) data. The % *positive correlation* column indicates the percentage of eye movements that correspond with patients looking less frequently into areas of reduced visual field sensitivity. The *standard deviation* and *direction from FVF observers* provides a measurement for the difference between patient and FVF distributions (see text for details). Subject 4 was the only patient that did not show a positive trend between fixation frequency and visual sensitivity*.

*Indicates subjects that did not have a distribution significantly different from FVF subjects based on the χ^2^ test.

### Stimuli

**Figure 1:**
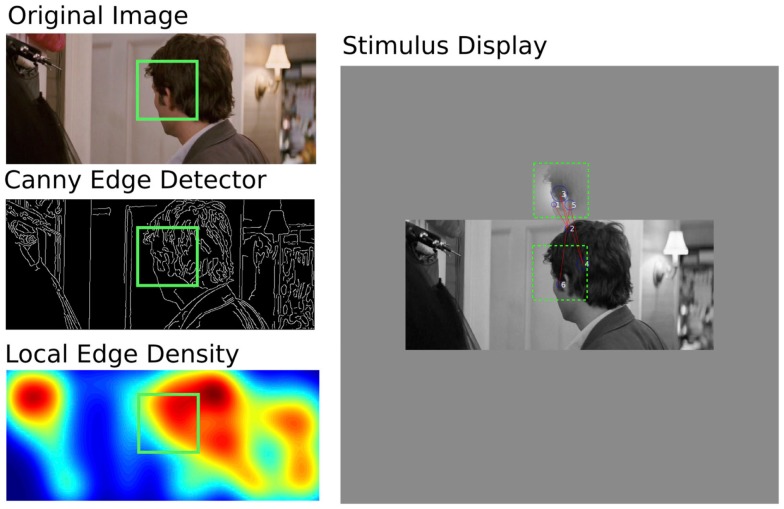
**Illustration of stimulus generation and example of typical eye movements**. Left Column – Stimulus Generation: (middle) a Canny Edge Detector located the edges in a source image (top). A Gaussian weighting function (σ*_x,y_* = 2°) was used to determine the mean number of edges in an area the size of the search target. This avoided the selection of targets with too few (e.g., blank regions of wall or sky) or too many (e.g., grass or blinds) features for localization. The green square (not shown in the experiment) illustrates the location of the search target selected with this method. Right Column – Display and Eye Movements: the target was placed in a window above the search image, its mean luminance (50 cd/m^2^) and rms contrast (0.2%) did not match the original in order to force a structural search. Fixations are depicted as blue circles, whose diameter represents fixation duration. Saccades are depicted as red vectors (not shown in the experiment). Example data are from Subject 5. Subjects were asked to locate the patch within the image and to click a mouse cursor on its location.

Observers performed a monocular visual search task that required them to locate a search target in a natural image. A screen shot of a typical stimulus from one trial is depicted in Figure 1. The red lines (saccades) and blue circles (fixations) overlaid on the image show representative eye movements made by one subject and were not displayed in the presentation of the stimulus. Stimuli were presented on a 21′′ Samsung Sync master CRT monitor at a resolution of 1152 × 864 (subtending 36° × 27°) with a refresh rate of 75 Hz and a mean luminance of 50 cd/m^2^. Observers viewed the display from a distance of 57 cm, and head position was stabilized using a chin rest. Patients wore spectacles for their best corrected vision. Eye movements were recorded from the eye with the most severe visual field loss in PVFL patients. FVF observers completed the task with a randomly selected eye. The unused eye was occluded with an eye patch.

Stimulus images were randomly selected from a database of over 85,000 frames derived from the Hollywood film *27 Dresses*. This database contains images representative of a broad range of commonly experienced natural/urban scenes, including a variety of indoor and outdoor settings, objects, faces, people, and animals. Figure 1 shows an example of how each stimulus image was created. For each trial, an image was selected at random and scaled to a mean luminance of 50 cd/m^2^ and a global root mean square (RMS) contrast of 0.2 (approximately 100% Michelson contrast). The search image was displayed at its source resolution (853 × 356 pixels) and subtended 26.66° × 11.13° on the monitor. Even with the search target 4° above the image, this stimulus is smaller than the area of a 54° × 48° Humphrey Visual Field. However, it is important to note that any fixations away from the center of the stimulus will bring the stimulus to the edge of the visual field, essentially doubling the viewing area to 53° × 22°. Furthermore, many of the PVFL patients had scotomas close to the fovea, which would occlude part of the display at almost any fixation location.

A smaller search target, subtending 4° × 4° (128 × 128 pixels) was extracted from a pseudo random location on the image, subject to the constraint that the area contained a typical density of edge features to avoid the problem of blank target patches (e.g., areas of sky that lack identifiable features) or highly textured target patches (e.g., areas of grass that cannot easily be discriminated from other densely textured patches). To allow us to make a target-selection based on edge density, prior to data collection, we computed the edge density (total number of edges divided by the total number of pixels per frame) of all images in the database. Edges were estimated using the Canny operator (Canny, 1986) accessed with the “edge” operator in Matlab (Mathworks, Ltd). The mean and standard deviation of this distribution was used to estimate the typical edge density of the database. A random deviate from this distribution was selected for each trial, and an area in the search image matching this edge density was selected as the search target. This process ensured that the search target contained unique identifiable features whose density varied from trial to trial. The mean luminance of the search target was fixed at 50 cd/m^2^ to match the background, and the RMS contrast of the search target was fixed at 0.2. This ensured that the local luminance and contrast of the search target differed from the values of the corresponding patch in the natural image, forcing the observer to locate the search target based on its spatial structure rather its brightness or contrast. The search target was presented within a Gaussian window (σ*_x,y_* = 2°) immediately above the center of the search image. A green central fixation point was presented between image presentations to provide a standard initial fixation location for each trial.

Participants were instructed to freely view the search image with the goal of identifying the location of the target. Once they had located the target, they indicated its position by moving a mouse-controlled cursor to the target and clicking either button. The time from stimulus onset to the mouse click was recorded as the search duration each trial. This response initiated the next trial. No time constraint was enforced. The task was repeated 30 times during a session. We considered asking observers to fixate the target when they detected it instead of making a mouse-based response; however, this method could trigger a false positive each time a subject fixated near the target without necessarily identifying it. Indeed, observers frequently glanced on or near the target area before the mouse response, as if to check whether alternative locations were a better match to the target. It is also possible that observers could have detected the target without directly foveating it. There were comparable levels of comorbidity in the groups (arthritis, etc.), so it is unlikely that dexterity differences between PVFL and FVF subjects may have affected motor response times.

### Analysis of eye movement data

Eye positions were collected during search trials using a 50-Hz Cambridge Research Systems Video Eye Tracker with spatial precision of 1.0°. At the start of each session, a nine-point calibration was performed with the supplied software. Eye tracking data were processed offline with software custom written in Matlab (Mathworks, Ltd). The raw eye positions from each trial were imported as a series of *x*, *y* screen coordinates sampled every 20 ms. The eye tracker flagged unsuccessful attempts to record eye position, typically during a blink or when fixation fell outside the eye tracker’s field of view, and we discarded trials in which the total proportion of untracked eye movements exceeded 50%. Across all subjects, an average of 20% of trials were rejected. In the remaining trials, untracked eye positions were linearly interpolated between tracked data because deleting untracked data would have introduced large, abrupt gaps between tracked data points. In the PVFL group, an average of 84% of the eye positions were successfully tracked on included trials, compared with 78% for the FVF group.

Changes in eye position (i.e., eye movement vectors) were computed from the change in *x* and *y* position between samples. The mean and standard deviation of all eye movements were calculated for each trial. Eye movements were classified as either saccades or fixations. A saccade was identified by the minimum of two possible criteria: an eye movement either whose speed exceeded 30°/s or whose speed exceeded the mean plus 2 SDs of the eye movement speed for the trial. The first criterion is commonly adopted in eye movement research; however, we found that the latter criterion was required to allow for inter-subject oculomotor variation (Cornelissen et al., 2005; McIlreavy et al., 2010). Eye positions between saccades were classified as fixations, which would encompass changes due to microsaccadic eye movements falling below the previous criteria. Within a fixation, the mean and standard deviation of all eye positions were computed to identify a single center of fixation and a measure of fixation stability, respectively.

## Results

### Search time, fixations, and saccades

**Figure 2:**
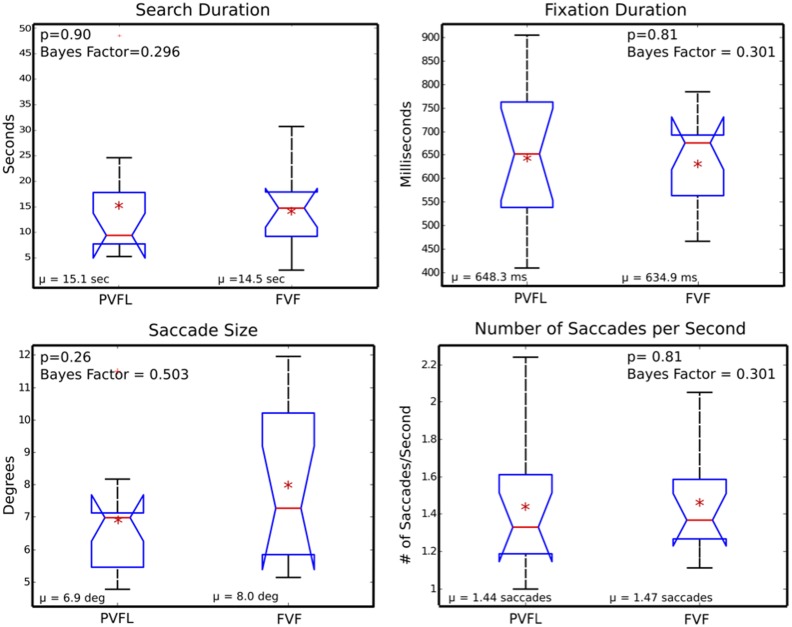
**Oculomotor summary measures**. Box plots of search duration, fixation duration, saccade size, and number of saccades per second, averaged across peripheral visual field loss (PVFL) patient and full visual field (FVF) groups. The red line indicates the median for each distribution and the edges of the box display the 25th and 75th percentile. The red asterisk indicates the mean of each distribution and a red cross marks outliers. Comparison intervals are shown with notches. Whiskers display the minima and maxima of the distributions (excluding outliers). None of the parameters were significantly different between PVFL and FVF groups (*p* values and Bayes Factors shown inset). Bayes factors are listed in the form alternative/null.

Figure 2 shows box plots for search duration, fixation duration, saccade size, and number of saccades per second between PVFL and FVF groups. Search duration and number of saccades per second were averaged across the 30 trials for each subject. Saccade size and number of fixations were collapsed over all trials for each subject and averaged to produce a single estimate. All saccades larger than the screen size were excluded during this comparison, which resulted in 0.7% of PVFL saccades and 1.2% of FVF saccades eliminated. Figure 2 depicts the distributions of the means of these parameters for each subject. The differences between groups were not statistically significant in any of the four measures.

The data show a clear null result for search duration, fixation duration, and saccades per second (*p* > 0.8). In addition to performing a two-sample *t*-test, we completed Bayes hypothesis testing and found all four parameters favored the null hypothesis (Rouder et al., 2009). Based on these bayes factors, the saccade size comparison shows anecdotal evidence for the null hypothesis, while the other three parameters show substantial evidence that there was no difference between the two groups (Wetzels et al., 2011). Bayes factors are listed along with *p* values from standard hypothesis testing in Figure 2. Given that search duration, fixation duration, and saccades per second are mutually dependent, it is not surprising that these parameters follow a similar trend. Although the sample size for this comparison is small, a *post hoc* power calculation (considering our effect size and a power of 0.8) required an impractically high sample size of 900 participants for significance, additionally the median search time was actually longer for FVF patients in direct contradiction of the experimental hypothesis.

### Distribution of saccade directions

**Figure 3:**
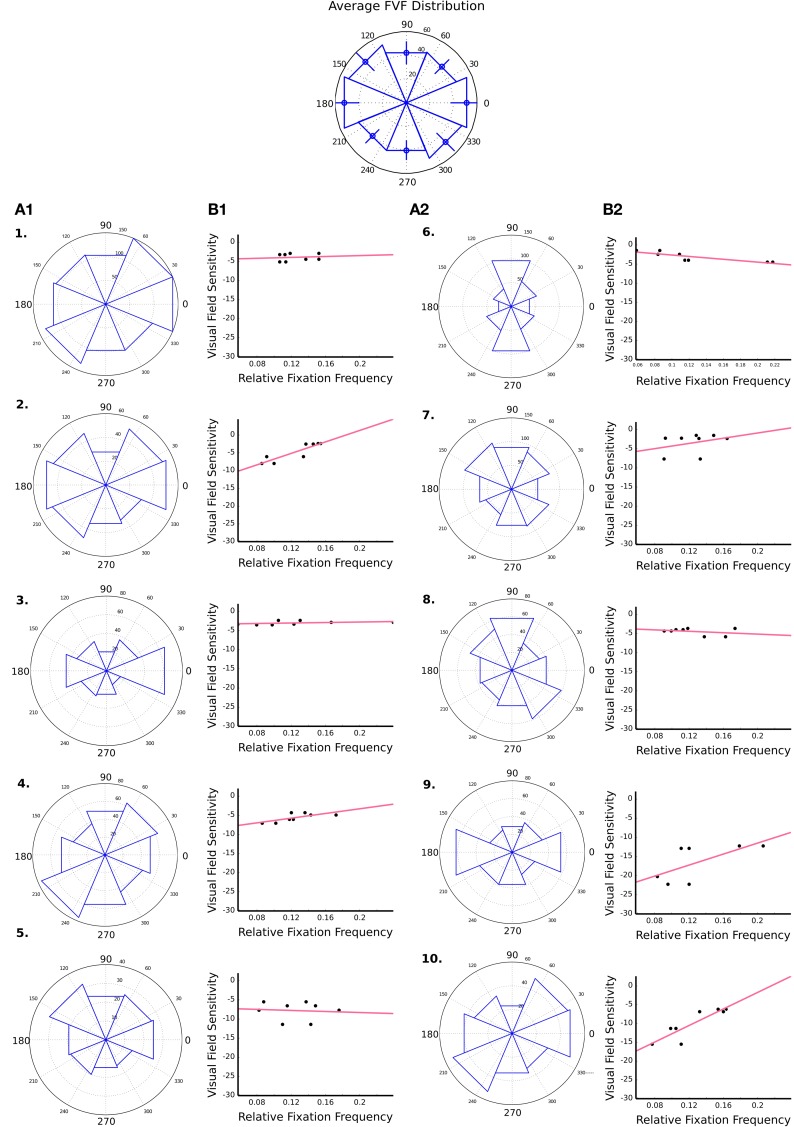
**Binned saccade direction distributions and visual field sensitivity**. The Columns A1 and A2 depict angle histograms for the distribution of saccade directions over all trials for each of the 10 PVFL patients. The FVF group histogram is centered above and is representative of a mean distribution for all FVF participants over all trials, with error bars to depict the standard error. Next to each histogram is a scatter plot of binned visual field sensitivity and binned saccade direction frequencies (eight bins of 45° each) for each PVFL patient. The red line represents the best-fit regression line.

The distributions of saccade directions across all trials are shown in Columns A1 and A2 of Figure 3. The histograms group the distribution of directions into eight evenly spaced bins, each spanning 45°. The top row depicts the mean distribution for FVF participants, error bars depict the standard error, and the following rows (labeled 1–10) depict data for each PVFL patient individually. A χ^2^ test showed that eye movements in FVF subjects were not evenly distributed. FVF observers made significantly more eye movements along horizontal axis than diagonal or vertical axis. A jackknife statistical method (Miller, 1974) and χ^2^ analysis showed that 6 out of 13 FVF participants had a significantly different saccade distribution from the mean FVF distribution (the mean calculation across all FVF observers was not significantly different from the mean with the inclusion of only those participants not significantly different, *p* = 0.6).

In order to correct for the anisotropy of eye movement directions, the mean direction distribution of all FVF observers was used to estimate the expected *a priori* distribution of eye movement directions for an unaffected observer, and a χ^2^ test was used to detect differences in the frequency of eye movement directions between PVFL patients and FVF observers. For each PVFL patient, a χ^2^ test compared the patient’s observed frequency of eye movements in each direction to an expected frequency based on the FVF distribution. After Bonferroni correction for multiple comparisons (i.e., eight bins), the test resulted in a significant difference in the frequencies of eye movements for 8 out of the 10 PVFL patients. *P* values are listed in Table 1.

### Relationship between eye movements and visual field defects

Figure 3 Columns B1 and B2 show the binned saccade directional data used in the χ^2^ analysis plotted against visual field sensitivity for each PVFL patient. The 54 values of visual field sensitivity are taken from the pattern deviation plot on the Humphrey Visual Field test. These values were grouped into eight evenly spaced bins (each spanning 45° around central fixation). The mean sensitivity value at each of those bins was then plotted against the relative saccade frequency to that bin location for each patient. The relative saccade frequency was calculated by dividing the number of saccades to each bin location by the total number of saccades for that patient. A positive correlation between visual field sensitivity and the PVFL relative frequency would indicate a patient looked less frequently into areas of the visual field known to be less sensitive. Six out of 10 PVFL patients showed a modest trend toward this positive correlation, but none of the comparisons were significant.

**Figure 4:**
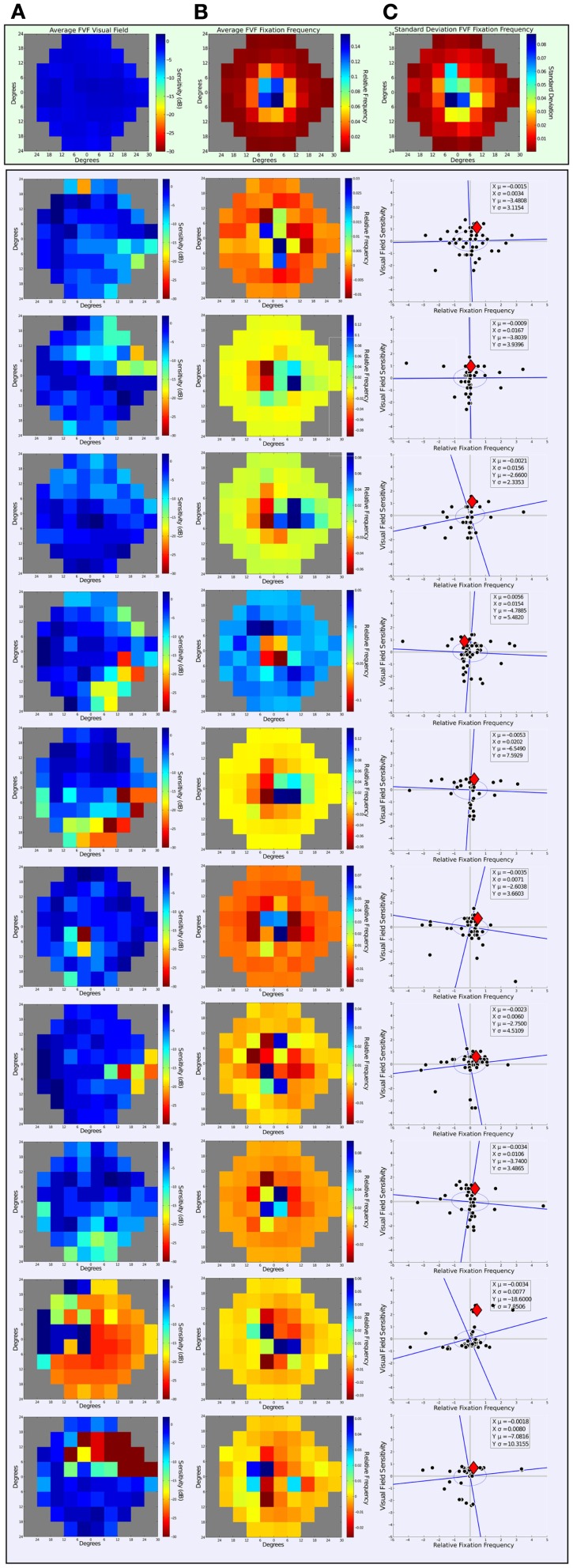
**Visual field sensitivity and fixation landing points**. The top rectangle contains data from the FVF group and the larger bottom rectangle contains data from each of the 10 PVFL patients. Column A shows the heat maps of visual sensitivity from Humphrey Visual Field test pattern deviations. Data collected from right eyes have been flipped for comparison purposes. Column B contains the relative frequency distribution of eye movements over all trials normalized to the center of visual field space. Frequencies are displayed as differences relative to the averaged FVF reference distribution. The top row of Column C includes a heat map of the standard deviation for fixation frequency across the 13 FVF observers to represent the variability across their distributions. Column C (rows 1–10) shows a point-wise comparison of visual field sensitivity and relative fixation frequency. The data are normalized as z-scores. The red diamond represents the location of a normally sighted observer (a summary estimate from the FVF group data).

Figure 4 Column A shows patterns of sensitivity across the visual field depicted as heat maps (where “hotter” areas depict areas with a higher deviation from normal). The top row shows the averaged data for FVF subjects, and Column A2-10 show the individual data for each PVFL subject. The Figure was created from each individual’s most recent pattern deviation plot on the Humphrey Visual Field test. Pattern deviation thresholds were binned into a matrix representative of the patient’s visual field. Each bin on the heat map spans 6° in *x* and *y* direction to create a visual field space spanning 54° horizontally and 48° vertically. All visual fields are normalized to the “left” eye field; specifically, visual fields of subjects tested with the right eye were horizontally flipped to facilitate comparison of deficits across subjects.

Figure 4 Column B shows the relative frequency distribution of eye movements for each subject across all trials. The top row shows the average data for FVF subjects and Column A2-10 show the individual data for each PVFL patient. The top row of Column C additionally includes a heat map of the standard deviation for fixation frequency across the 13 FVF observers to represent the variability in their distributions. The 54-bin heat maps span 54° horizontally and 48° vertically and are thus directly comparable to the representation of visual field sensitivity in Column A. The distribution of saccades over all trials was normalized to the center of visual field space for each subject. For each saccade, the start point is positioned at the center of the 54-bin heat map. The endpoints were summed to generate a saccade frequency to a visual field location represented by each bin. This fixation frequency was then expressed as the proportion of eye movements to each location to account for the difference in total number of eye movements among subjects. This was achieved by dividing the frequency of eye movements to each bin by the total number of eye movements over all trials for each observer. Even in FVF subjects, the frequency of eye movement endpoints is not uniform. This anisotropy means that we cannot simply examine the frequency of fixations to a given location without considering how frequently FVF observers make eye movements to this location. We therefore express the proportions of eye movement endpoints for the PVFL patients relative to FVF subject data in order to compare how the observed eye movements differ from those expected in an unaffected observer. The difference between each PVFL patient’s eye movement frequency and the average of the 13 FVF frequencies (Figure 4 Row 1 Column B) was calculated at each bin location to determine the frequency distribution relative to the FVF reference group. Negative values correspond to locations in the visual field where the PVFL patient made fewer eye movements relative to FVF observers and positive values correspond to where the patient made more eye movements relative to the FVF reference.

Next we correlated relative visual field sensitivity with relative eye movement frequency at all visual field locations. Before looking at the relationship between visual field sensitivity and fixation frequency distributions, we used jackknife statistics (Miller, 1974) to eliminate outliers in both the visual field sensitivity and fixation frequency distributions for each patient. An outlier was defined as any datum that altered the standard deviation of the original distribution by two or more standard deviations of the original distribution. With this criterion, 5.1% of all data points (including visual sensitivity and eye movement frequency) were eliminated. This procedure did not significantly affect any calculations made that compared visual field sensitivity and eye movement frequency data (i.e., percent of positively correlated points and standard deviation measures).

In order to compare visual field sensitivity and fixation frequency on similar scales, we converted both variables to *z*-score distributions by dividing each datum by the standard deviation for that variable. Figure 4 Column C shows a point-wise comparison of the normalized scores, with visual sensitivity on the *y*-axis and relative fixation frequency on the *x*-axis. After plotting the normalized scores in this new space, we fit an ellipse to the data. We defined the semi-major axis of the ellipse by the regression line of the *z*-score distributions (visual sensitivity versus fixation frequency) and the orthogonal served as the semi-minor axis. We then calculated the distance from each data point to both the semi-major and semi-minor axis of the ellipse. The standard deviations of each of these distance distributions defined the limits of the two axis of the ellipse on the semi-minor and semi-major axis, respectively. The red diamond on the plots represents the origin of the non-normalized data in this new space, i.e., the location of the normally sighted FVF observer relative to the visually impaired observer. For 9 out of the 10 patients, the center of the normalized distribution fell left and below this reference point. This means that visually impaired patients made fewer eye movements into locations of reduced visual sensitivity (patient four’s data fell to the right of this reference point, indicating more eye movements were made to areas with less sensitivity). Based on these normalized distributions, only 2 out of 10 patients (Patient 3 and 9) were individually significantly different from the FVF cumulative distribution (i.e., the center of the ellipse fell more than 2 SDs from the reference point of normal sensitivity and derived normal fixation frequency). It should also be noted that Patient 9 exhibited the most severe visual field loss, indicating any novel pattern of viewing may surface in more severe cases of PFVL.

The plots in Column C depict a modest positive trend between visual field sensitivity and fixation frequency in most PVFL subjects. As expected, most of the visual sensitivity data fall in the lower half of the figure, indicating that most visual field locations had lower sensitivity in patients. Most of the data were also in the left side of the figure, indicating that PVFL patients made fewer eye movements than FVF reference subjects to areas of their visual field shown to have lower sensitivities than the controls housed in the automated perimetry database. The percentage of points from the non-normalized distributions that were positively correlated (i.e., negative relative sensitivities at locations of negative frequency differences and positive relative sensitivities at locations of positive frequency differences) was calculated for each of the 10 patients. These values are listed in Table 1. Nine out of the 10 PVFL patients tested had more than 50% positively correlated points. Even though this trend was not significant, all patients made many eye movements into visual field locations with impaired vision. These eye movements could not have been directly initiated by feed-forward salience-based responses, unless they were based on responses from a previous fixation.

## Discussion

Patients with bilateral peripheral field loss report difficulties with everyday tasks that involve hazard or target detection, while driving or walking, especially on stairs (Nelson et al., 1999; Viswanathan et al., 1999; Haymes et al., 2007; Ramulu, 2009). Many visual tasks require subjects to search complex and cluttered scenes for target(s), and the present study identifies differences in eye movements that may contribute to these difficulties. While there was no systematic change in oculomotor dynamics (i.e., the size, frequency, and duration of saccades and fixations), there was a difference in the *direction* of eye movements. In 9 out of 10 cases, there was a trend for patients to make fewer than expected eye movements into the locations of local visual field defects while searching.

The present results suggest that PVFL patients tend to make fewer eye movements (i.e., saccades) to locations where stimuli are less visible due to the presence of a scotoma. Our findings complement earlier work that examined eye movements of patients with severe PVFL (7°–15° field) as they freely walked a course (Vargas-Martín and Peli, 2006). Compared to normally sighted subjects, the spatial extent of horizontal scanning was reduced in PVFL patients. The authors proposed that lack of visible peripheral targets resulted in the restricted spatial range of eye movements. This interpretation aligns with our observation that some patients did not compensate for field loss by making additional eye movements to impaired locations, but instead, ignored affected areas. This observation is similar to a study with multi-resolution display; Loschky and McConkie (2002) found that observers made fewer saccades into the surrounding peripheral region when it was of lower resolution. However, it should be noted that another subsequent study examining eye movements in a similar population of PVFL patients reports no significant effects of reduced visual field size on oculomotor behavior. This difference may have been due to more salient stimuli and greater task-demands in the second study (Luo et al., 2008).

Crabb et al. (2010) examined eye movements of glaucomatous patients viewing movies that depicted typical driving scenes and found no significant differences in fixation locations between patients and controls. This may be due to the fact that the bivariate contour ellipse (BCE) analysis the authors used only considered the image space, in contrast to our analysis that compared FVF subjects and PVFL patients in visual field space (i.e., how the image fell on particular locations on the retina). Their study does, however, report individual examples of patients following a different pattern of viewing from controls, more specifically regarding instances when patients were not aware of particular aspects of the stimuli. Based on our hypothesis that field loss may affect eye movement behavior in predictable ways, it was pertinent that we analyzed eye movements in the context of visual field space to account for variation in peripheral retinal sensitivity.

There are at least two factors that are currently thought to determine eye movement behavior. Feed-forward models predict that fixations are driven by “feature salience” estimated from the activity of low-level sensory mechanisms (Nothdurft, 1993; Wolfe, 1994; Itti and Koch, 2001; Mazer and Gallant, 2003). Successive fixations may additionally involve a computation of the maximum information gained from potential fixation locations, which can lie between locations of maximum salience (Najemnik and Geisler, 2005). According to this view, eye movement behavior in a visual search task can be guided by the visibility of salient stimuli in the periphery. Additionally, eye movements can be driven by high level, task-demand factors that may maximize task-relevant information (Land et al., 1999; Foulsham and Underwood, 2007; Henderson et al., 2007; Einhäuser et al., 2008). The present visual search task was specifically designed to minimize the role of top-down factors by selecting image patches of randomized contrast that rarely contained recognizable objects. Our observers were required to search for an image patch based on matching spatial features, with minimal dependence on object recognition and scene properties. In agreement with a previous study, our findings are not consistent with an *exclusively* feed-forward model, since observers frequently made eye movements into areas of vision loss (Henderson et al., 1997). While some observers made fewer eye movements into areas of impaired vision (those with positive slopes in Figure 3), others were more likely to make an eye movement into an unsighted location (those with negative slopes in Figure 3). The former trend is consistent with exclusively feed-forward models, while the latter contradicts this approach.

It was critical that the spatial distribution of eye movements was plotted relative to each fixation center to create a reference comparable to the visual field for each patient. The retinal image falling on the peripheral visual field during the visual search task changed with each new saccade – thus image locations that fell within a scotoma on one fixation fell on a different, potentially sighted retinal locus on other fixations. This means that it may be difficult to find a significant impairment on visual search tasks unless individual eye movement directions and landing points are examined. We created a 2D space that depicted the distribution of eye movements for the entire data set over all trials in order to make a more robust judgment on any directional bias observed in PVFL patients. Additionally, the 2D space allowed us to make a direct comparison between eye movement distribution and the patient’s visual field.

Point-wise comparisons between field loss and eye movements are made based on assumptions derived from the averaged FVF dataset. All fixation frequencies for PVFL patients are expressed relative to the reference subjects who did not demonstrate visual field loss on two or more standard automated perimetry tests. The reference data showed that small eye movements were more likely than large and indicated a relatively uniform distribution of eye movements to all directions of the visual field, with a slight increase in movements made in the horizontal direction, consistent with previous observations from experiments probing visual search within natural scenes (Crane and Kelly, 1983; Tatler and Vincent, 2009). Although there have been reports of contrary findings of a preponderance of saccade direction perpendicular to the horizon (for rotated natural scenes), that study did not require subjects to perform a task (Foulsham and Kingstone, 2010). The horizontal bias is a consequence of the asymmetric shape of the binocular visual field and may have been exaggerated by the nature of the presented stimulus, which spanned a greater width than height. We examined the difference in the spatial distribution of eye movements from the reference data to account for the fact that many saccades made by PVFL patients were similar in size, resulting in a clustering of endpoint changes around the center of each matrix. This measure helped to uncover subtle differences in directional distribution of eye movements relative to the center of the visual field and was important in accounting for any bias in distribution based on the stimulus presentation. In an attempt to minimize top-down factors, the search target was displayed directly above the search image, which may have biased the distribution of eye movement directions in the vertical direction. We did not observe a recognizable pattern of eye movements in the direction of the target because each eye movement changed the relative location of the target image and most eye movements were within the large search image. Furthermore, the results were expressed relative to FVF patients who would be similarly biased.

We looked at several other aspects of eye movement behavior during search: total search duration, fixation duration, saccade size, and number of saccades showed no difference between PVFL patients and FVF subjects. This finding is consistent with some previous reports showing no significant effect of PVFL under some, but not all conditions (Luo et al., 2008; Smith et al., 2011). The simplest explanation for this is that while a peripheral target may fall within a scotoma on one fixation, it may fall in sighted locations on other fixations, reducing the overall effect of local visual field defects on oculomotor parameters or search times. However, the comparison of search duration is limited by our measurement method, which required subjects to move and click the mouse to identify the target location. The time between locating the target and clicking the mouse was not measured and is an additional source of response noise. Although there is no reason to assume any motor differences between groups, this additional response noise could have affected the null result in this particular comparison. We chose to use the time of mouse click rather than the first mouse movement because there were large differences in mouse movement between observers, with some moving the mouse several times throughout the trial. Similarly, we could not use the first gaze-point on the target because gaze points were frequently on or near the target before other locations and the location response.

The null oculomotor results contrast with Crabb et al. (2010) who found that glaucomatous patients made significantly more saccades and fixations per second when viewing driving scenes and for similar measurements made with artificial tunnel vision (Cornelissen et al., 2005). This inconsistency may be attributed to the difference in stimuli (e.g., natural images versus optotypes, or moving versus static images). Magnocellular retinal ganglion cells, thought to be impaired in glaucoma patients, are selective detection of moving structures (Dandona et al., 1991). Given that a moving scene will engage different neural populations, this could potentially lead to clearer differences in oculomotor behavior with PVFL (Falkenberg and Bex, 2007). Additionally, there may be strategic differences for moving versus static images; for example, potential targets are only present for a short period in a driving scene, so there is greater time pressure as objects move in and out of the field of view. In short, characterization of visual search may require both static and moving stimuli.

Smith et al. (2011) showed significant differences between search times for patients and controls when they search for a single object within an everyday photograph, but no significant difference in performance when they searched for an upright Landolt C in an array of distracters (rotated Landolt Cs). Subjects performed the task binocularly – mirroring more everyday conditions – but we examined monocular search in order to examine the impact of the loci of scotomas. Our results are applicable to patients with bilateral PVFL, as well as monocular patients who have lost all useful vision in a single eye. Both of these types of patients can experience significant affects from field loss and are common among glaucoma patients (Crabb et al., 1998). Directional analysis of oculomotor behavior, with an emphasis on binocular search performance, may provide better insight into how monocular peripheral deficits affect eye movement behavior while performing daily activities.

Visual search is an everyday task and a better understanding of eye movements while engaging in such a task could improve the rehabilitation of optic-nerve-based PVFL patients. Patients with PVFL experience deficits in a broad range of daily activities (Ramulu, 2009), that may be related to an inability to adequately process their environment with peripheral vision (Gutierrez et al., 1997; Nelson et al., 1999; Viswanathan et al., 1999; Jampel et al., 2002; Noe et al., 2003; Friedman et al., 2007; Haymes et al., 2007). It is likely that on each fixation, useful information for visual search falls into a scotoma, which reduces the total information available for saccade planning for each fixation. Based on the feed-forward models of visual search, we speculate that if patients could learn to make eye movements in the direction of their field loss, any information *gained* could be beneficial in interacting with the complex environments of everyday activities. Alternatively, additional eye movements to the loci of defects may hinder overall search performance by requiring additional eye movements to sub-optimal locations. There may be little potential for novel information gain due to the redundant structure of natural scenes. Perhaps our finding that patients make similar eye movements (i.e., number of saccades, fixation duration, and saccade size) to FVF observers in visual search may be indicative of the fact that neither compensatory strategies nor avoidance of scotoma locations are optimal in improving search performance.

General parameters of visual search (i.e., search duration and number of saccades) failed to show any significant difference between subjects with and without PVFL. A more specific analysis of eye movement directions revealed patients’ show a biased directional distribution that does not directly relate to visual field loss. We found that some patients do not compensate for visual field loss. This insight may be useful in pursuing new opportunities for search-based rehabilitative training and outcomes metrics for patients with PVFL.

## Conflict of Interest Statement

The authors declare that the research was conducted in the absence of any commercial or financial relationships that could be construed as a potential conflict of interest.

## Supplementary Material

The Supplementary Material for this article can be found online at http://www.frontiersin.org/Perception_Science/10.3389/fpsyg.2012.00472/abstract

## Appendix

**Table:**

PVFL subjects	Classification	Acuity	Age	DX	Medications
1	Glaucoma	20/30	83	Overweight, hypertension, atrial fibrillation, lyme disease, chronic renal impairment, hiatal hernia, gastroesophageal reflux disease, colitis, gout, cervical disc disease, sciatica	*Alphagan*, asacol, *azopt*, calcium citrate, lisinopril, lorazepam, multivitamins, *ocuvite*, pravachol, vitamin d3, warfarin sodium, *xalatan* (may 2010)
2	Glaucoma	20/25	82	Type 2 diabetes, atrial fibrillation, fuchs corneal dystrophy	*Acular*, *combigan*, *cosopt*, coumadin, *dorzolamide*, metoprolol, *xalatan*
3	Glaucoma	20/20	82	Osteoporosis, glaucoma, cataract, hypercholesterolemia, HTN, ppd+/cxr+/−, gastritis	*Alphagan*, atorvastatin, colace, fosamax, hydrocodone, *lumigan*, norvasc, omeprazole, potassium chloride, *cosopt*, turns
4	Glaucoma	20/20	58	Hypertensive disorder, seasonal allergies	Caltrate, cardizem, losartan, mvi, simvastatin, *timolol*, *travatan*
5	Glaucoma	20/20	55	Type 2 diabetes, cardiac catheterization, hypertension hypercholesterolemia, asthma, obesity, coronary artery disease, angioedema urticaria	Aspirin, coreg, epipen, flonase, lipitor, metformin, vitamin d3, *xalatan*
6	Glaucoma	20/40	74	Rheumatoid arthritis, depression, glaucoma, cirrhosis of liver, lymphadenopathy, scoliosis, kidney stone, osteopenia, hypertension	Aspirin, coreg, epipen, flonase, lipitor, metformin, vitamin d3, *xalatan*
7	Glaucoma	20/20	67	Rosacea, hypertension, glaucoma, elevated cholesterol	Clindamycin, diovan, hydrochlorothiazide, k-dur, metrogel, multivitamins, oxycodone, pravachol, timoptic, vitamin d3, xalatan
8	Glaucoma	20/25	79	Hypertension, elevated cholesterol, glaucoma, prostate cancer	Alphagan, aspirin, cosopt, flomax, hydrochlorothiazide, labetalol hcl, lipitor, lisinopril, *lumigan*, multivitamins, norvasc, trazodone
9	Glaucoma	20/25	58	Hypercholesterolemia, glaucoma, h/o myocardial infarction, placement of stent in coronary artery	*Alphagan*, aspirin, *cosopt*, crestor, lisinopril, multivitamin, plavix, *travatan* z, tricor
10	Glaucoma	20/20	45	Strabismus, glaucoma	Allegra, levoxyl, naproxen, nortriptyline hcl, *patanol*

**FVF subjects**	**Classification**	**Acuity**	**Age**	**DX**	**Medications**

1	Suspect	20/30	67	Hypercholesterolemia, onychomycosis, glaucoma, right long apical scarring, alcoholism, smoking, hypertension, pulmonary nodule, asthma	*Acular*, hydrochlorothiazide, lidex 0.05% cream, *lumigan*
2	Suspect	20/30	60		Simvastatin
3	Suspect	20/20	64	Prostate cancer	Multivitamins
4	Suspect	20/25	44	Arthritis, seasonal allergies, hernia repair, depression	Allegra, atenolol, diovan, imitrex, *trusopt* 2%, *xalatan*
5	Suspect	20/30	83	Hypertension, superficial phlebitis, raynauds, glaucoma, smoker, hypothyroidism, osteoporosis	*Alphagan*, aspirin, levoxyl, lisinopril, olux foam, prednisolone 1%, synalar, *xalatan*
6	Suspect	20/30	57	H/O TOB 85, low hdl, drusen, colon, atrial fibrillation	Cipro, colace, dronedarone, flagyl, flomax, omeprazole, vancomycin hcl, *xalatan*
7	Suspect	20/30	60	Hypothyroidism	Aspirin, desonide, levoxyl, *timolol maleate* 0.5%
8	Suspect	20/20	49	Depression, osteopenia, hypothyroidism, uterine leiomyoma, anemia	Calcium, desipramine, ferrous sulfate, levothyroxine sodium, lorazepam
9	Suspect	20/40	82	Hearing loss, depression, h/o adenoma, arrhythmia, mitral valve prolapse, tinea	Aspirin, colace, miralax
10	Suspect	20/20	67	Osteoporosis, depression, gastroesophageal reflux disease, chronic interstitial cystitis	Astelin, estradiol tab, omeprazole, progesterone cream, *xalatan*
11	Suspect	20/20	70	Seasonal allergies	Albuterol inhaler, flonase, lovastatin, multivitamin, nabumetone, prilosec, viactiv
12	Suspect	20/20	74	Hypertension, diabetes mellitus, arthritis	Digoxin, diltiazem, glipizide, isoniazid, lisinopril, multivitamins, plaquenil sulfate
13	Suspect	20/20	66	Hypertension, hypothyroidism, colonic polyps, uterine fibroids	*Betagan 0.25%*, hctz, levoxyl, mvi, triamterene 37.5
